# Morality and management: an oxymoron? fNIRS and neuromanagement perspective explain us why things are not like this

**DOI:** 10.3758/s13415-020-00841-1

**Published:** 2020-10-29

**Authors:** Michela Balconi, Giulia Fronda

**Affiliations:** 1 Department of Psychology, Catholic University of the Sacred Heart, Milan, Italy; 2 Research Unit in Affective and Social Neuroscience, Department of Psychology, Catholic University of the Sacred Heart, Milan, Italy

**Keywords:** Moral decision-making, fNIRS, RTs, Fairness, Managerial

## Abstract

The neuroscience interest for moral decision-making has recently increased. To investigate the processes underlying moral behavior, this research aimed to investigate neurophysiological and behavioral correlates of decision-making in moral contexts. Specifically, functional Near-infrared spectroscopy (fNIRS) allowed to record oxygenated (O2Hb) and deoxygenated (HHb) cerebral hemoglobin concentrations during different moral conditions (professional fit, company fit, social fit) and offers types (fair, unfair, neutral). Moreover, individuals’ responses to offers types and reaction time (RTs) were considered. Specifically, from hemodynamic results emerged a difference in O2Hb and HHb activity according to moral conditions and offers types in different brain regions. In particular, O2Hb increase and a HHb decrease were observed in ventromedial and dorsolateral prefrontal cortex (VMPFC, DLPFC) for fair offers in professional fit condition and in superior temporal sulcus (STS) for unfair offers in social fit condition. Moreover, an increase of left O2Hb activity in professional fit condition and in right VMPFC for unfair offers in company fit condition was observed. In addition, from behavioral results, an RTs increase in company and social fit condition for fair and unfair offers emerged. This study, therefore, shows the behavioral and neurophysiological correlates of moral decision-making that guide moral behavior in different context, such as company one.

## Introduction

In recent years, moral decision-making has been a topic of great interest in different disciplinary fields (Tobler et al. 2008). Specifically, moral decision-making can be defined as a complex process that involves any decision, judgment, or evaluation of actions’ acceptability and fairness within the moral domain (Garrigan et al. 2018; Rilling and Sanfey 2010).

Considering its complexity, moral decision-making appears to be mediated mainly by emotional and rational processes (Greene et al. 2004; Loewenstein et al. 2001). The former is related to the evaluation of socially relevant stimuli as right or wrong; the latter consists of rational and deductive reasoning processes concerning the possible costs and benefits associated with moral decisions (Brand et al. 2006; Greene et al. 2004). During moral decision-making, therefore, individuals are not only “emotional agents” but also “rational agents” who formulate evaluations to maximize decisions’ costs and benefits to obtain material and social rewards (Izuma et al. 2008). In light of this multidimensionality, the initial psychological interest for moral decision-making has been gradually extended to all everyday decision-making contexts, as demonstrated by an increased interest in companies’ moral behaviour. In particular, the investigation of processes underlying moral fair or unfair decision-making in the company context is crucial, because it can produce positive or negative social consequences, in terms of consumers, employees, and community’s health, safety, and welfare, and it has a significant effect on the organizational quality of the culture (Minas et al. 2014; Woolley et al. 2007). In this regard, the perception of fairness can be considered as an adaptive mechanism that individuals use within a psychosocial framework of cooperation and justice (Bereczkei 2015). On the contrary, the perception of unfairness appears to be associated with negative emotions that lead individuals to reject unfair offers (Gaertig et al. 2012). Recently, the interest in the perception of unfairness has increased, mainly within social contexts (Wu et al. 2011, 2012), leading to an “inequality aversion,” which occurs when individuals perceive inequalities, preferring equity (Strobel et al. 2011; White et al. 2014). Unfairness aversion has been mainly investigated by some studies that have shown how unfair offers’ rejection provide greater activation of striatal regions that appear to be involved in reward mechanisms (Strobel et al. 2011; White et al. 2014) and individuals’ gratification (De Quervain et al. 2004).

Furthermore, the perception of fairness and unfairness is mediated by empathy, which plays an important role in the morality field (Eisenberg 2000; Van Vugt et al. 2011). Specifically, the empathy concept is defined as the implementation of a behavioural response lead by an individual to another to induce a well-being condition (Batson 2008). In particular, empathy is mediated by two interconnected processes: a cognitive one, which consists of adopting the other person’s perspective in a given situation, and an emotional one, which consists of feeling sympathy, compassion, and tenderness towards others that characterizes interpersonal and prosocial relationships, strengthening social interactions, and cooperation (Pavlovich and Krahnke 2012; Pizarro and Salovey 2002). Moreover, within moral context, empathy favours a better contemplation of the possible decisions’ implications, consequences, and responsibilities regarding other individuals’ well-being, through the evaluation of the social benefits of choices (Dietz and Kleinlogel 2014; Mencl and May 2009). On the contrary, the lack of empathy is generally associated with less guilt and consideration for the moral implications of choices, resulting in more correlated to utilitarian decision-making (Dietz and Kleinlogel 2014; Mencl and May 2009). Especially in contexts that require to make decisions that can have social consequences, such as in a company context, it is therefore very important to observe how empathic behaviour influences moral decision-making.

Indeed, the recent interest in company moral decision-making has led to the investigation of all individual and situational variables underlying moral behaviour (Minas et al. 2014; Woolley et al. 2007), considering the processes and brain structures involved in moral decision-making not previously investigated by some studies that have focused the attention only on the application of different theoretical guides in the management of daily decisions (Boatright 2014; Velasquez and Velazquez 2002). Indeed, many social networks appear to be involved in the feeling of belonging and reciprocity towards a specific condition (Harvey et al. 2010) or other individuals (Rilling et al. 2002) implicated in moral decisions.

The individual and situation variables underlying moral decision-making were explored by tasks of social decision consisting of monetary paradigms developed in the field of game theory (Sanfey 2007; Stallen and Sanfey 2013). These paradigms were found to be very useful in optimizing the choice behaviour of the player (Krajbich et al. 2009; Schiebener and Brand 2015), but they did not investigate the emotions underlying decision-making, which provide information on individuals’ interpersonal sphere and emotional processing and responses (Wagner et al. 2012). Moral reasoning and moral decision-making can be considered complex constructs requiring the involvement of different brain networks. In particular, the neural correlates underlying moral behaviour have been investigated mainly with the use of functional magnetic resonance imaging (fMRI) throughout tasks requiring the expression of a moral judgment related to the adequacy of individuals’ actions or the evaluation of visual scenes (Avram et al. 2013; Boccia et al. 2017; Garrigan et al. 2016; Schaich Borg et al. 2008; Yoder and Decety 2014). Specifically, as demonstrated by previous studies (Greene et al. 2001, 2004), the fMRI allowed the exploration of the emotional, cognitive, and utilitarian processes involved in moral decision-making. Indeed, some previous studies have used fMRI to explore how individuals’ brains react to the others’ judgment of positive, negative, or neutral actions (Plitt et al. 2015), observing the cerebral circuits and the large brain networks underlying moral behaviour that appear to be involved in the theory of mind (ToM) (Hein and Singer 2008; Garrigan et al. 2016; Fumagalli and Priori 2012; Jack et al. 2012; Moll et al. 2008; Plitt et al. 2015). Some of these cerebral regions, such as the dorsolateral prefrontal cortex (DLPFC), the orbitofrontal cortex, the ventromedial prefrontal cortex (VMPFC), the anterior cingulate cortex (ACC), the precuneus, the temporoparietal junction, the parietal lobe, and the superior medial prefrontal cortex (SMPFC) are most involved in objective and moral reasoning (Amodio and Frith 2006; Balleine et al. 2007; Boccia et al. 2017; Chang et al. 2002; Fuster 2000; Greene and Haidt 2002; Jack et al. 2012; Pascual et al. 2013; Poldrack et al. 2001; Tanaka et al. 2006), providing a specific contribution to moral behavior. In particular, the VMPFC is particularly involved in the control of the emotional responses associated with processes of moral decision (Garrigan et al. 2016; Young and Dungan 2012) related to individuals’ moral standards and social values (Eres et al. 2018; Moll et al. 2002a, 2002b).

The involvement of this brain area in the emotional processes underlying the moral judgment is highlighted by several studies (Blair et al. 2006; Fumagalli and Priori, 2012; Garrigan et al. 2016; Marazziti et al. 2013; Raine and Yang 2006).

In addition to the VMPFC, the orbitofrontal cortex appears to be involved in the processing of salient information related to the possible sense of guilt or moral judgment experienced by individuals (Eres et al. 2018; Molenberghs et al., 2014, 2015; Moll et al., 2002a). Instead, the temporal-parietal junction and the precuneus appear to be implicated in the attribution of mental states related to others’ moral judgment (Eres et al. 2018; Young and Koenigs, 2007). In addition to these cerebral regions, that are particularly implicated in moral processes, other studies have shown the involvement of some brain networks coactivated during moral decision-making. In particular, the cortical midline structures (CMS), comprising the DLPFC, the VMPFC, and the cingulate cortex, are particularly implicated during moral judgment and decision-making (Damasio 2010; Han et al. 2016; Northoff 2004).

In this regard, to investigate the moral decision-making processes, the present research used a monetary task, consisting of a modified version of the Ultimatum Game (UG). Specifically, this task asks individuals of a company context to make decisions related to different daily contexts of moral choice (professional fit, company fit, and social fit context) and types of offers (fair, unfair, or neutral). This paradigm allowed the exploration of the influence of fairness and unfairness perception in company moral decision-making by proposing ecological contexts of choice. Specifically, in each of the three contexts, a sum of money was proposed in three different situations: professional fit for a job done together with a colleague; company fit for the introduction of some company’s benefits; and social fit to help the care of a colleague’s relative financially. The offers proposed for the attribution of money could provide an advantage or a disadvantage to the respondent or concern a proposal of an equal attribution between the proponent and the respondent.

In addition, to investigate the emotional and cognitive processing underlying moral decision-making, brain activity was considered during an ecological moral decision-making task. From the neuroscientific perspective, fairness and unfairness in moral perception were mediated by specific brain regions (Tabibnia et al. 2008). In particular, the former is associated with the activation of bilateral insula, left hippocampus, and left lingual gyrus (Rilling et al. 2008). The latter results in activating more the DLPFC and the anterior cingulate cortex, implicated in objectives’ control and in the detection of cognitive conflicts. Some studies also have observed the role of the VMPFC and the DLPFC in moral decision-making and their implication in moral judgment and in emotional processes underlying choices with possible gains and social benefits (Hare et al. 2010), showing a fundamental role, especially for the DLPFC, in the evaluation of choices’ short- and long-term benefits (Levy and Glimcher 2011; Sanfey 2007).

In this regard, the growing interest in neurosciences and the use of neuroscientific tools to investigate the processes underlying moral decision-making have allowed researchers to deeply observe the neurophysiological correlates of moral behaviour (Naqvi et al. 2006). In the present study, the changes of cerebral blood oxygenation were continuously recorded with the use of functional near-infrared spectroscopy (fNIRS) (Ferrari, Giannini, Sideri, & Zanette, 1985; Franceschini et al. 2003; Jobsis, 1977; Villringer, Planck, Hock, Schleinkofer, & Dirnagl, 1993).

Specifically, fNIRS is a neuroimaging technique which, thanks to its portability and movement’s tolerance, turns out to be useful for the investigation of the brain responsiveness associated with specific brain regions during complex sensory, motor, and cognitive tasks (Balconi et al. 2015a; Balconi and Vanutelli 2016; Leff et al. 2011). Indeed, as demonstrated by previous studies, fNIRS was found to be a useful tool for the recording of moral behavior, because it allows to monitor changes continuously in oxygenated (O2Hb) and deoxygenated (HHb) hemoglobin in the cerebral cortex, also useful on clinical patients (Dashtestani et al. 2018; Franceschini et al. 2003). For this reason, it has been used in several studies designed to investigate the effects of personal incentives on moral decisions and to investigate moral behavior in psychopathological conditions (Dashtestani et al. 2019; Strait and Scheutz 2014). Furthermore, to observe the cognitive-behavioural mechanisms underlying different choices conditions and offer types, the individuals’ performance was recorded in terms of offers’ options (number of accepted or rejected responses) and RTs accepting or rejecting of the proposed offers. Indeed, as demonstrated by previous studies, RTs responses provide information about the cognitive load and the utilitarian process underlying moral decision-making (Krajbich et al. 2009; Youssef et al. 2012).

RTs measurement, indeed, has always been an indicator that has provided answers on the information processing speed (Kyllonen and Zu 2016). Specifically, as proposed by the “memory drum” theory (Henry and Rogers 1960; Klapp 2010), the most complex answers require more time for the greater load of information stored in memory, differently from simpler ones.

Therefore, the present study was designed to observe the brain and cognitive behavioural mechanisms underlying different contexts of choice and types of offer. Specifically, regarding hemodynamic activity, firstly, we assumed to observe a different O2Hb and HHb haemoglobin activation related to different choice conditions. In particular, we expected to observe a different leftward versus rightward asymmetry of frontal neural activity related to different conditions of choice, according to the dual systems model of neural signatures of affective experience, which reports an increased left-hemispheric activity in response to more positive conditions and more right hemispheric activity in response to more negative and aversive conditions (Balconi et al. 2015b). Moreover, we assumed to observe a different cortical O2Hb and HHb activity of specific frontal areas in relation to different choice conditions (professional fit, company fit, social fit) and offer types (fair, unfair, neutral). In particular, it was expected to reveal a specific activation of the prefrontal areas (such as DPLFC and VMPFC), which are more implicated in moral judgment, intrinsic moral sense, and empathic mechanisms, during the presentation of fair offers that propose maximum personal advantages compared with others. Significant differences between brain areas’ activation also are expected for personal/professional fit condition in comparison with the company or social ones.

Specifically, the company fit condition is considered less emotionally engaging than the other two conditions, with relevant effects on empathic behaviour and responsiveness. In addition, for what concern behavioural data, we assumed to observe different options of responses (accept or reject) and RTs to various offers types (fair, unfair, and neutral) and conditions of choice (professional fit, company fit, and social fit condition). In particular, we expected to observe an increased number of accepted offers in fair and neutral offers compared to unfair ones in professional and social fit conditions compared with company one, which does not involve advantages or disadvantages for people, but for material objects.

Moreover, we expected to observe an increase of RTs in response to more conflictual and critical choice contexts (such as the company or social fit conditions compared to professional fit one). In contrast, we expected to observe a decrease of RTs to offers’ presentation in the professional fit condition, which implies less cognitive effort regarding personal interests and a sort of “facilitation effect” of choice.

## Method

### Participants

Eighteen managers from an Italian company (age M = 43,71; SD = 11,56, 8 women) took part in the research. For the participants’ recruitment, the following exclusion criteria (age <18 years; the presence of psychiatric or neurological pathologies, presence of cognitive deficits, and clinical history of neurological or psychiatric disorder) were used. All participants took part in the study after signing the informed consent. The research was conducted following the principles and guidelines of the Helsinki Declaration and was approved by the local ethics committee of the Department of Psychology of the Catholic University of Milan.

### Procedure

The subjects were placed in a dimly lit room in front of a computer monitor at a distance of 70 cm. Participants were asked to perform a task administered through the E-Prime 2.0 software (Psychology Software Tools, Inc., Sharpsburg, PA). Specifically, the task, which consists of a modified version of the UG, proposed three different randomized moral conditions of choice (professional fit, company fit, and social fit). In particular, the task required two players: the proponent (different according to the context of choice) and the respondent (the individual who performed the task) to attribute a sum of money. The proposer decided how to attribute the sum of money, and the respondent could decide whether to accept or reject the proposed offer. If the respondent decided to refuse the offer, no player would take money.

In the professional fit condition, it was proposed individuals to attribute a sum of money (1,000 euros) to a colleague for an extra-remunerated job done together. Specifically, the professional fit condition proposes individuals to accept or reject three offers related to the attribution of a sum of money as compensation for a job done together with a colleague. For each offer rejected by individuals, neither of them gets the money.

Instead, in the company fit condition, it was proposed individuals the attribution of a sum of money (1,000 euros) for the inclusion of some company benefits in their work environment. Specifically, the company fit condition proposes individuals to accept or refuse to attribute a part of a company bonus to help to increase company benefits, such as the construction of a corporate residence, a gym, and other benefits.

Finally, in the social fit condition, it was proposed that individuals divide a sum of money to support a colleague’s relative with health problems financially. Specifically, the social fit condition proposes that individuals accept or refuse to donate a part of their company bonus to help the relative of a colleague with health problems financially.

For each condition (professional fit, company fit, and social fit), 15 scenarios were presented. The different choice conditions were presented in three blocks that lasted approximately 15 minutes. At the end of the scenario presentation, three different offers of attribution of money (fair, unfair, and neutral) were proposed. Each offer was repeated randomly 15 times.

In particular, the fair offers proposed a favourable attribution of money for the respondent (60% respondent and 40% bidder). The unfair offers proposed an unfavourable attribution of money for the respondent (40% respondent and 60% bidder). Finally, the neutral offers proposed an equal attribution of money for both subjects (50% respondent and 50% bidder). Participants could accept or reject the proposed offer by pressing the “1” and “0” keys on the computer keyboard. For each offer, subjects were reminded that if they refused, they would not get the money.

**Fig. 1:**
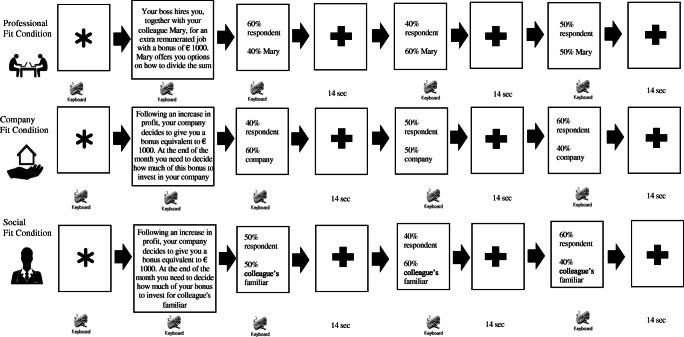
Research experimental procedure

The trial structure included: an initial blank screen, the presentation of the choice’s scenario, the presentation of the first offer, a blank interstimulus (14 sec), the presentation of the second offer, a blank interstimulus (14 sec), the presentation of the third offer, a blank interstimulus (14 sec). Specifically, the three offers (fair, unfair, and neutral) were presented individually on the screen until the participant decided whether to accept or reject the offer proposed to record the response times. Moreover, participants were not given a defined time interval to decide whether to accept or reject the proposed offer. Before the experiment started, 15-min simulation of the paradigm was presented to participants as a familiarization phase, following the same structure (Fig. 1).

### fNIRS recording and analyses

**Fig. 2:**
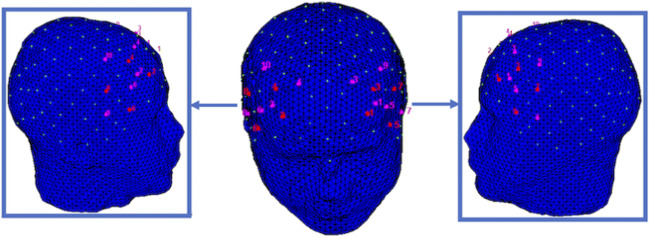
fNIRS Montage from the frontal and lateral sides head view. The emitters (violet) were placed on positions F7, F3, F4, F8, FC5, FC6, AF3h, and AF4h, while the detectors (red) were placed on the following positions: F5, F1, F2, F6, FT7, FC3, FC4, FT8, AF6h, AF5h

The fNIRS measurements were conducted with the NIRScout system (NIRx Medical Technologies, LLC, Los Angeles, CA). For the recording of the hemodynamic activity, 18 optodes (8 light sources and 10 detectors) were placed on the prefrontal cortex (PFC) and temporal regions. Specifically, the sources were positioned on the following positions: F7, F3, F4, F8, FC5, FC6, AF3h, AF4h, while the detectors were placed on the following positions: F5, F1, F2, F6, FT7, FC3, FC4, FT8, AF6h, AF5h. Therefore, the following channels (Ch) were acquired: Ch1 ​​(AF3h-AF5h), Ch2 (AF4h-AF6h), Ch3 (AF3h-F1), Ch4 (AF4h-F2), Ch5 (F7-F5), Ch6 (F8-F6), Ch7 (F7-FT7), Ch8 (F8-FT8), Ch9 (F3-AF5h), Ch10 (F4-AF6h), Ch11 (F3-F5), Ch12 (F4-F6), Ch13 (F3-F1), Ch14 (F4-F2), Ch15 (F3-FC3), and Ch16 (F4-FC4), Ch 17 (FC5-FT7), Ch18 (FC6-FT8), Ch19 (FC5-FC3) and Ch20 (FC6-FC4) (Fig. 2). Specifically, the sources and detectors were positioned at a distance of 30 mm on the subject's scalp using an fNIRS cap (international system 10/5), and a near-infrared light of two wavelengths (760 and 850 nm) was used. The variations in the concentration of O2Hb and HHb were recorded continuously with the NIRStar acquisition software. The signals obtained from the 20 fNIRS channels were measured with a sample rate of 6.25 Hz, analysed, and transformed with the nirsLAB software (v2014.05, NIRx Medical Technologies LLC, 15 Cherry Lane, Glen Head, NY). The raw data from the individual channels were digitally filtered through 0.01-0.3 Hz bandpass. This filter procedure allowed to suppress the heartbeat. The computation of hemodynamic signals from the raw data (modified Beer-Lambert law) was obtained, according to their wavelength and location, which resulted in values for the changes in the concentration of oxy and deoxygenated hemoglobin for each channel, which was scaled in mmol∗mm.

Furthermore, a linear-phase FIR filter on respiration (0.3 Hz), providing the symmetric-impulse-response, was employed (Balconi et al. 2020; Naseer and Hong 2013, Naseer, Hong, and Hong, 2014). The average concentration of O2Hb and HHb activity was calculated for each condition (professional fit, company fit, and social fit) and offer type (fair, unfair, and neutral) by creating specific indices, such as the difference between baseline averages (N; m1) and condition (m2) divided by the standard deviation (s) of the baseline: d = (m1 - m2) / s. Successively blocks averages were exported and further analyzed using a different statistical processing software (Statistical Package for Social Science, SPPS).

As final step, data were reimported (for NIRSLab processing software) to produce the figures. Ten different channel positions were calculated for both left/right homologous side: Ch1 and Ch2 positioned on the left and right antero-frontal cortex; Ch3 and Ch4 positioned on the left and right ventromedial frontal cortex; Ch5 and Ch6 positioned on the left and right fronto-lateral cortex; Ch7 and Ch8 positioned on the left and right fronto-temporal cortex; Ch9 and Ch10 positioned on the left and right medial frontal cortex; Ch11 and Ch12 positioned on left and right dorsolateral frontal cortex; Ch13 and Ch14 positioned on left and right dorsolateral and medial frontal cortex; Ch15 and Ch16 positioned on left and right fronto-central cortex; Ch17 and Ch18 positioned on left and right fronto-centrotemporal cortex; Ch19 and Ch20 positioned on left and right anterior temporal cortex.

## Results

### Data analysis

Two sets of ANOVA analyses were performed with respect to behavioral (offers responses options and RTs) and neurophysiological (fNIRS: O2Hb, HHb) dependent measures. For both sets of ANOVA tests, the degrees of freedom were corrected using Greenhouse–Geisser epsilon when appropriate. Post hoc comparisons (contrast analyses) were applied to the data. Bonferroni correction was applied for multiple comparisons for both behavioral and fNIRS data.

Recent advances in multichannel fNIRS allow wide coverage of cortical areas while entailing the necessity to control family-wise errors (FWEs) due to increased multiplicity. Conventionally, the Bonferroni method has been used to control multiple comparisons. Type I errors (false positives) can be strictly controlled.

The Bonferroni-based methods are especially stringent in controlling Type I errors of the most activated channel with the smallest *p* value. Although other methods can be applied (e.g., effective multiplicity (*M*_eff_)—derived from the eigenvalues of correlation matrices—to maintain a balance between Types I and II errors), Bonferroni method for the present data, also based on a quite limited number of channels (n = 20), was considered adequate. In addition, the normality of the data distribution and the normality assumption was preliminarily tested and supported (kurtosis and asymmetry tests).

### Behavioral data

By using the E-prime Software, individuals’ options responses (number of accepted or rejected proposed offers) and RTs related to individuals accepting or rejecting offers responses were obtained. Then, a repeated measure ANOVA was applied to offers responses and RTs dependent measures, with Condition (3, professional fit, company fit, and social fit), and Type (3, fair, unfair, and neutral) as repeated factors.

**Fig. 3:**
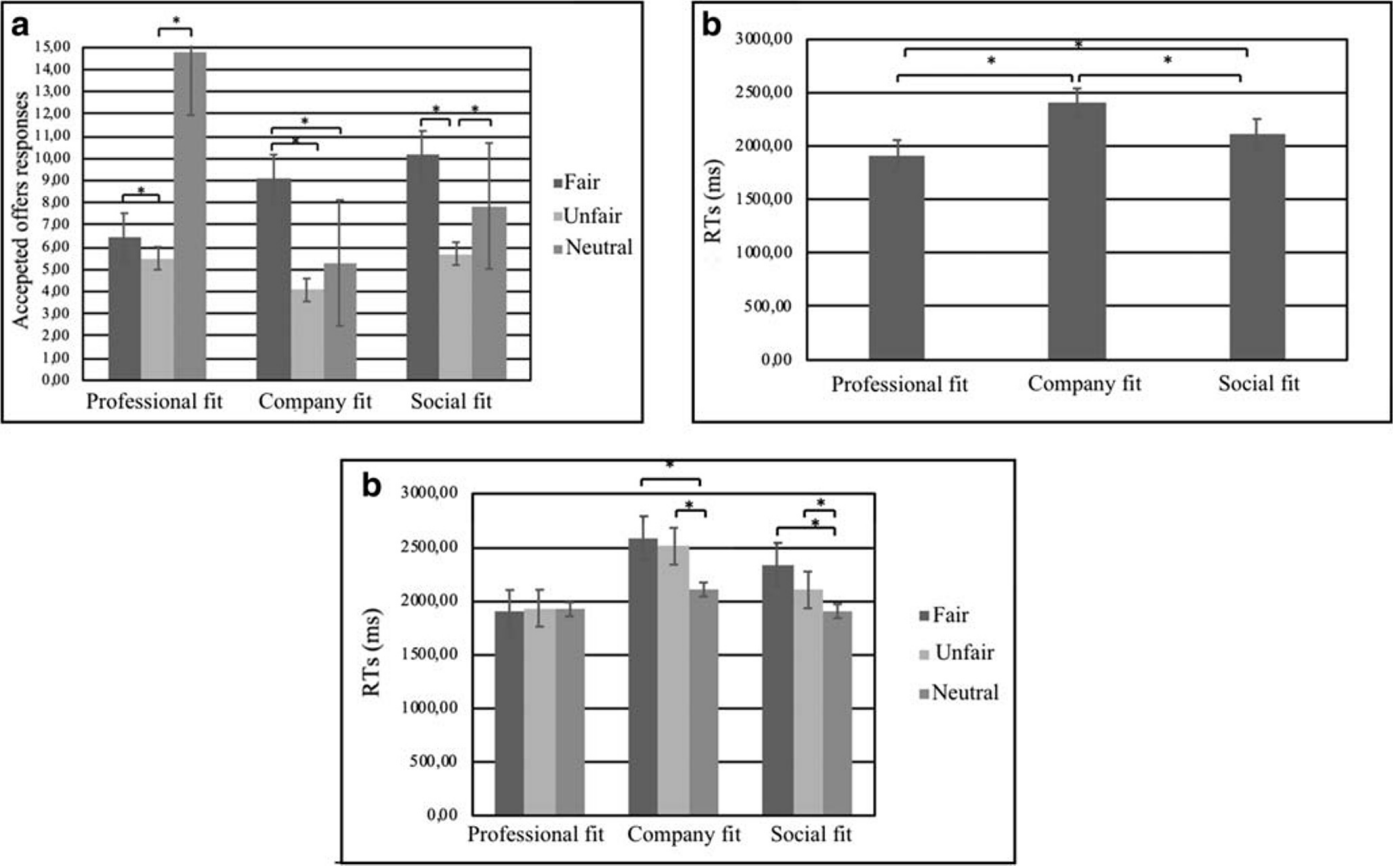
Offers response option and RTs. (**a**) Number of accepted responses to fair, unfair, and neutral offers in professional fit, company fit, and social fit conditions. (**b**) RTs offers responses in professional fit, company fit, and social fit conditions. (**c**) RTs offers responses to fair, unfair, and neutral responses in professional fit, company fit, and social fit conditions

For individuals’ options of response ANOVA revealed a significant interaction effect for Condition X Type (F[2,17] = 8.88, *p* < 0.01, η^2^ = 0.30). In particular, as revealed by post hoc comparisons an increase of accepted responses was found in fair and neutral offers compared to unfair in professional fit (respectively F[2,17] = 10.11, *p* < 0.01, η^2^ = 0.34) and social fit (respectively F[2,17] = 9.13, *p* < 0.01, η^2^ = 0.32) conditions; in fair offers compared with unfair and neutral ones in company fit condition (Fig. 3a).

For RTs, ANOVA revealed a significant effect for Condition (F[2,17] = 8.34, *p* < 0.01, η^2^ = 0.29). As revealed by post-hoc analysis, an increase of RTs was found in company fit condition compared with professional (F[1,17] = 7.89, *p* < 0.01, η^2^ = 0.28) and social fit ones (F[1,17] = 8.02, *p* < 0.01, η^2^ = 0.29). Moreover, an increase of RTs was observed in social fit condition (F[1,17] = 9.08, *p* < 0.01, η^2^ = 0.31) compared with professional fit one (Fig. 3b). Finally, ANOVA revealed a Type X Condition interaction effect (F[4,17] = 10.65, *p* < 0.01, η^2^ = 0.34). Specifically, as revealed by post-hoc comparisons, an increase of RTs was found for fair and unfair offers compared to neutral ones in company fit condition (respectively F[1,17] = 8.22, *p* < 0.01, η^2^ = 0.29; F[1,17] = 7.88, *p* < 0.01, η^2^ = 0.27) and social fit condition (respectively F[1,17] = 7.09, *p* < 0.01, η^2^ = 0.28; F[1,17] = 8.90, *p* < 0.01, η^2^ = 0.29; Fig. 3c).

### fNIRS data

The statistical analyses were applied to the dependent variable (d indices) for O2Hb and HHb measures. Two repeated measures ANOVAs were applied to O2Hb and HHb concentration levels with Condition (3), Type (3), Lateralization (2, left/right), and Channel positions (n = 10) as repeated measures.

**Fig. 4:**
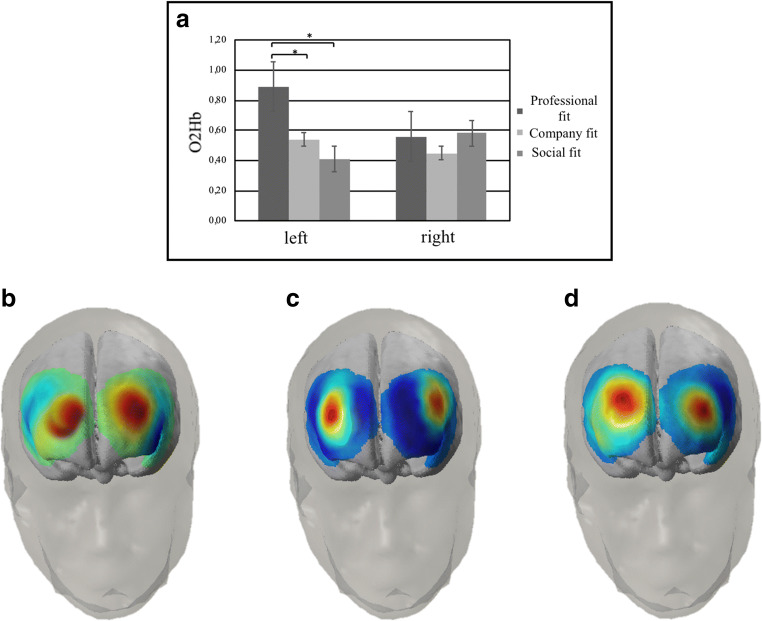
O2Hb and HHb activity. (**a**) Concentration of O2Hb activity in the cerebral left and right side in professional fit, company fit, and social fit conditions. (**b**) O2Hb and HHb SPM t-statistic map of hemodynamic activity in channel positioned over ventromedial and dorsolateral frontal cortex for fair offers in professional fit condition block. (**c**) O2Hb and HHb SPM t-statistic map of hemodynamic activity in channels positioned over anterior temporal cortex for unfair offers in social fit condition block. (**d**) O2Hb and HHb SPM t-statistic map of hemodynamic activity in channel positioned over the right ventromedial frontal cortex for unfair offers in company fit condition block

About O2Hb, ANOVA show significant interaction effects for Condition x Lateralization (F[2,17] = 9.76, *p* < 0.01, η^2^ = 0.29), Condition x Type x Channel positions (F[16,17] = 12.33, *p* < 0.01, η^2^ = 0.36) and Condition x Type x Channel positions x Lateralization (F[16,17] = 9.54, *p* < 0.01, η^2^ = 0.31). Specifically, post-hoc comparisons revealed an increase of brain activity (O2Hb) in professional fit condition more than company fit (F[1,17] = 8.23, *p* < 0.01, η^2^ = 0.29) and social fit one (F[1,17] = 10.54, *p* < 0.01, η^2^ = 0.33) in the left side channel positions compared with the right ones (Fig. 4a). In addition, an increase of activity in channels positioned over ventromedial frontal cortex and dorsolateral frontal cortex more than the other positions (for all comparisons *p* < 0.01) was found for fair offers in professional fit condition (Fig. 4b). Moreover, an increase of activity was found for unfair offers in social fit condition in channels positioned over anterior temporal cortex more than the other positions (for all comparisons *p* < 0.01; Fig. 4c).

Finally, an increase of O2Hb activity was found for unfair offers in company fit condition in the channel positioned over the right ventromedial frontal cortex compared with left one (F[1,17] = 12.08, *p* < 0.01, η^2^ = 0.36; Fig. 4d).

About HHb, Condition x Lateralization (F[2,17] = 8.09, *p* < 0.01, η^2^ = 0.29), Condition x Type x Channel positions (F[16,17] = 9.54, *p* < 0.01, η^2^ = 0.32), and Condition x Type x Channel positions x Lateralization (F[16,17] = 9.32, *p* < 0.01, η^2^ = 0.31) significant interaction effects were found. Specifically, post-hoc comparisons revealed a decrease of frontal HHb for fair offers in professional fit condition in channels positioned over ventromedial frontal cortex more than the other positions (for all comparisons *p* < 0.01) and in dorsolateral frontal cortex more than the other positions (for all comparisons *p* < 0.01; Fig. 4b). Moreover, a decrease of frontal HHb activity was found for unfair offers of social fit condition in channels positioned over anterior temporal cortex more than the other positions (for all comparisons *p* < 0.01; Fig. 4c).

Finally, a decrease of HHb was found for unfair offers of company fit condition in channel positioned over the right ventromedial frontal cortex compared with the left one (F[1,17] = 8.15, *p* < 0.01, η^2^ = 0.29; Fig. 4d).

## General discussion

The present study was designed to investigate possible differences in individuals’ cerebral and behavioural responses concerning different conditions (professional fit, company fit, and social fit) and offers (fair, unfair, and neutral offer) of moral decision-making. The results allowed us to report the following main points: 1) The evaluation of others’ benefits and advantages, even to the detriment of their advantages, varies depending on the decision’s context (professional fit condition compared to social or company fit conditions); 2) the cognitive “cost” for moral decision-making varies as a function of personal engagement (modulated by professional fit condition compared to social or company fit conditions); 3) the different contribution by specific brain areas implicated in more cognitive or emotional and empathic processes in response to moral conditions; 4) the specific effect of fairness as a critical factor able to modulate the subjective choices, with significant impact on both cognitive and neurophysiological level.

First, we have observed an increase of accepted options of response in fair and neutral offers compared to unfair ones in professional fit and social fit conditions, and in fair offers compared with unfair and neutral ones in company fit condition. This evidence shows that individuals are more willing to offer benefits fairly to someone or gain personal advantages in professional and social fit conditions, but only to derive personal benefits in company fit condition. This result could be due to the fact that in professional and social fit conditions individuals, therefore, would experience more empathic mechanisms with other individuals involved in the attribution of a sum of money compared with the company fit condition in which the decision concerns mainly working company. As shown by some previous research, empathic behaviour favours a better contemplation of the possible decision’s implications, consequences, and responsibilities regarding others’ well-being and allows them to evaluate costs or social benefits of choices (Dietz and Kleinlogel 2014; Mencl and May 2009).

Second, an increase in RTs in the company fit condition compared with professional and social fit was observed. This result could be related to the fact that the company fit condition compared with others requires a greater cognitive decision-making effort and a higher degree of uncertainty due to an assessment that does not directly concern one’s personal interests. In contrast, subjects were more likely to make more immediate choices (higher reduction of RTs compared with both social and company fit conditions) due to less complex cognitive processes and a higher direct engagement, in case of professional fit, which supports a more immediate ability to produce the moral decision. As demonstrated by previous studies, cognitively more complex processes require higher cognitive cost and resources that results to be associated with higher RTs. On the contrary, cognitively less complex processes are associated with faster RTs, because they require less information processing (Klapp 2010). Therefore, we may state that a sort of “continuum” from professional to social to company fit condition from less to more cognitive cost and mental resources consumption was observed across the task.

Regarding the fairness of the moral choices, from behavioural results emerged an increase of RTs for fair and unfair offers compared with neutral ones in the company and social fit conditions. This result could be due to the fact that in the company and social fit conditions, which do not directly involve individuals’ personal interest, neutral offers appear to be the most immediately acceptable options compared with fair and unfair ones, because it maintains an equilibrium, which may provide no advantages or disadvantages for anyone.

From the results of the hemodynamic data (O2Hb), we have observed a general increase of O2Hb activity in the channels positioned over the left hemisphere for the professional fit condition and in the channels positioned over the right hemisphere for the company fit condition. Specifically, this hemispherical asymmetry could be due to different emotional processing related to proposed conditions of choice. The professional fit condition could be perceived by individuals as more positive in terms of emotional engagement and personal interests, whereas the company fit condition could be perceived as more negative or less engaging by individuals. This finding is in line with previous results on the relationship between frontal hemispherical asymmetries and the valence of an emotion-laden experience. Indeed, as supposed by the dual systems model of the neural signatures of affective experiences (Balconi et al. 2015a), positive experiences and stimuli that induce approaching behaviours are typically associated with the activation of a left prefrontal system, whereas less positive experiences and stimuli that induce avoidance behaviours are typically associated with the activation of a right prefrontal system (Balconi and Mazza 2009, 2010; Harmon-Jones 2003).

In addition, considering both hemodynamic parameters (O2Hb and HHb measures), heterogeneous brain areas activation was revealed, depending on different choice conditions and offer types. As expected, significant modulations of O2Hb measures are consistently supported by the concomitant trends of modulation of the HHb parameter.

Specifically, in concomitance to O2Hb increasing, the concentration of HHb decreased in channels positioned over ventromedial and dorsolateral frontal cortex in correspondence to the professionally fit condition for fair offers, in channels positioned over anterior temporal cortex in correspondence to the social fit condition for unfair offers, and in channel positioned over the right ventromedial frontal cortex in company fit condition for unfair offers. More specifically, it emerged that during the presentation of professionally fit conditions fair offers induced an increase of O2Hb and a decrease of HHb in channels positioned over ventromedial and dorsolateral frontal areas.

Specifically, the greater activation of these channels might be associated to an increase of cerebral activity in these areas due to the fact that this choice condition, concerning individuals’ main interests, could activate brain regions involved in positive moral judgment, intrinsic moral significance, and in sharing mechanisms (Barriga et al. 2009; Marazziti et al. 2011; Tangney et al. 2007). Indeed, the ventromedial frontal and prefrontal cortex, as demonstrated by the activity of the ventromedial prefrontal cortex (VMPFC), appears to be more implicated in moral and emotional value attribution to personal events and in the empathic mechanisms activated when individuals fully identify himself with the proposed situation (D’Argembeau et al. 2008; Moll et al. 2008; Moll and de Oliveira-Souza 2007). Indeed, as demonstrated by previous studies, empathy appears to play a fundamental role within moral decision behaviour, because it favours an assessment of the possible consequences and implications of a decision considering mainly its social benefits (Dietz and Kleinlogel 2014; Mencl and May 2009).

In addition, the VMPFC area appears to be involved in the control of emotional responses involved in moral behavior and in the experience of prosocial feelings, such as guilt and compassion. Indeed, it has been shown that a reduction in prosocial behavior that often is associated with a deficit in the VMPFC involves the use of utilitarian choices in moral decision-making (Greene, 2007; Greene et al. 2001, 2004).

Furthermore, during fair offers presentation, in professional fit condition an increase of O2Hb and a decrease of HHb in channels positioned over the dorsolateral frontal areas was observed. In particular, as demonstrated by previous research, the greater activation of these channels might be associated with an increase of activity in these cerebral regions, which appear to be more implicated in the equity perception of decisions, in the evaluation of possible consequences of moral choices, and in the rejection of the unfair offers (Haidt 2001; Moll et al. 2003; Tangney et al. 2007).

Indeed, it has been shown that the activity of this cerebral portion area, including the dorsolateral prefrontal (DLPFC), is involved in the utilitarian judgment (Greene et al. 2004) and in the abstract reasoning, consisting in the analysis of costs and benefits (Glenn et al. 2009; Greene et al. 2001, 2004). Indeed, the role of DLPFC in rational cognitive control also has been shown by some studies that have observed how a reduced functioning of this brain region leads to a difficulty in utilitarian judgment, decreasing the refusal of unfair offers (Knoch et al. 2006; Zheng et al. 2018).

In contrast, in social fit condition specifically under unfair offers presentations, greater activation of O2Hb and a decrease of HHb activation was observed in channels positioned over the anterior temporal cortex. This result could be due to the fact that in social fit condition, which requires individuals to support a colleague’s sick relative, the increased activity of these channels might be associated with an increase of activity in those cerebral areas more involved in moral support, joint attention mechanisms, and pain sharing. Indeed, as underlined by previous research, the activity in the anterior temporal cortex, as in the superior temporal sulcus (STS) area, appears to be implicated in social perception, emotional elaboration, and social cognition processes (Allison et al. 2000; Greene et al. 2004; Harenski et al. 2010) and in others’ beliefs and intentions inferences (Allison et al. 2000), proving to be important in determining where other people’s emotions are directed (Marazziti et al. 2011). In particular, this area, in addition to being associated in the processes of social perception, also is implicated in the emotional and social cognition processes, activating above all in the moral dilemmas that affect one’s person compared with others (Greene et al. 2001, 2004; Harenski et al. 2008; Pascual et al. 2013).

Finally, from hemodynamic results, an increase of O2Hb and a decrease of HHb emerged in the channel positioned over the right portion of the ventromedial frontal cortex in company fit condition compared with others, during the presentation of unfair offers. Specifically, the increased activity of this channel might be associated with an increased activity in the right medial and frontal cortex, which as demonstrated by the activity of the right portion of VMPFC, appears to be more implicated in emotional negative value attribution (Marazziti et al. 2011). In light of this previous evidence, this result may be due to the fact that in this condition, which concerns the introduction of company benefits in the workplace, individuals evaluate different offers more rationally and perceive a greater sense of unfairness when disadvantageous offers are proposed even if this decision is toward an external object (such as company).

## Conclusions

The present study underlined the importance of understanding cerebral and behavioural correlates of individuals’ moral decision-making within a company context. In particular, this study provides an overview of cerebral and cognitive processes related to the evaluation of moral implications and fair and unfair consequences of moral decisions on personal and social interests, underlying how moral behaviour is influenced by individual and situational factors. Indeed, these results, on both cerebral and behavioural perspectives, offer an overview of the functional brain activation and the nature of the cognitive processes underlying moral decision-making. In this regard, we may remark that some areas may function as moderator of fairness in engaging personal condition (when personal interest are implicated), such as ventromedial and dorsolateral frontal areas. Conversely, other brain areas are able to mark and signal unfairness for choices not direct toward personal interests, which implicate the external world with higher social or business significance, such as more social or external (company) situations.

The present study showed that moral decision-making in the company context could be considered as a process of choice that represents a continuum between the evaluation of individual and social interests, highly affected by the subjective perception of the intrinsic advantages or disadvantages to themselves and others. Indeed, as demonstrated by the results of the present study, the evaluation of possible decisions advantages and disadvantages represents an important and complex process, especially when concern themselves or others interests. The former leads to greater activation of individuals’ different cerebral areas mainly involved in positive judgment, intrinsic moral sense, and empathy and sharing mechanisms. On the contrary, offers that do not concern themselves or other individuals but a more neutral object (such as the company) may be perceived as less personally relevant and may activate brain areas to connote (and remark) the disadvantageous condition (such as company unfairness). In contrast, the social fit condition may activate more “empathic” and intention attribution processes, which are required to discard unfair offers and to support fair ones. As demonstrated by the results of the present study, the evaluation of fair and unfair offers also is supported by different cognitive processes in terms of mental cost to decide, with an increased cognitive effort from the more personal perspective (professional ones) to the social condition until the external (company) condition.

Despite the innovativeness of the paradigm used for the investigation of moral decision-making, the present study has some limitations. The former concerns the necessity to adopt a multimethodological approach that allows to record not only cerebral correlates underlying moral decision-making, but also, for example, autonomic activation, more directly related to the emotional components of behaviour. The second concerns the use of a specifically modified version of the UG, with higher ecological value in comparison with the previous paradigm, but that is still linked to the economic (money-related) decision-making. In light of this evidence, therefore, in future studies, we could think of observing not only cerebral responses and cognitive measures, but also autonomic activity related to moral decision-making in different daily situations and type of offers to further explore moral behaviour. In addition, some technical aspects should be considered due to the fNIRS features in terms of data acquisition for depth-limitation. Therefore, we are aware that the present acquisition of the cortical activity may not include deeper-lying tissues in sulci. This limitation may prevent to specifically discuss the broader effect of experimental conditions on the cortical surface. In addition, due to imprecise nature of fNIRS with regard to anatomical location, cortical brain areas implicated in moral decision-making process should be better explored in future research in term of channel position/brain areas correspondence. Furthermore, in future studies, to better generalize and increase the power of the results, an ample sample size could be considered, although in the present research the presence of different conditions increases the reliability of the data collected.
